# A Case of Nongerminomatous Germ Cell Tumor of the Pineal Region: Risks and Advantages of Biopsy by Endoscopic Approach

**DOI:** 10.1155/2018/5106701

**Published:** 2018-03-11

**Authors:** Mauro Dobran, Davide Nasi, Fabrizio Mancini, Maurizio Gladi, Massimo Scerrati

**Affiliations:** Department of Neurosurgery, Umberto I General Hospital, Università Politecnica delle Marche, Ancona, Italy

## Abstract

A 21-year-old male was admitted to our department with headache and drowsiness. CT scan and MRI revealed acute obstructive hydrocephalus caused by a pineal region mass. The serum and CSF levels of beta-human chorionic gonadotropin (beta-hCG) were 215 IU/L and 447 IU/L, respectively, while levels of alpha-fetoprotein (AFP) were normal. A germ cell tumor (GCT) was suspected, and the patient underwent endoscopic third ventriculostomy (ETV) with biopsy. After four days from surgery, the tumor bled with mass expansion and ETV stoma occlusion; thus, a ventriculoperitoneal shunt was positioned. After ten months, the tumor metastasized to the thorax and abdomen with progression of intracerebral tumor mass. Despite the aggressive nature of this tumor, ETV remains a valid approach for a pineal region mass, but in case of GCT, the risk of bleeding should be taken into account, during and after the surgical procedure.

## 1. Introduction

Primary intracranial germ cell tumors (GCTs) are 3.1% of all primary brain tumors [1]; they affect pediatric population (mean age of 16.1 years) with a male : female ratio of 4 : 1 and are located in midline structures such as suprasellar and/or pineal region [2]; tumors contemporary located at suprasellar and pineal regions called synchronous GCT are 13% of all GCTs; these lesions are germinoma (60–80% of patients) with high sensitivity to radiotherapy [3], teratoma (18%), endodermal sinus tumors (7%), embryonal carcinomas (5%), and choriocarcinomas (5%). Teratoma is also divided into mature, immature, and anaplastic [4]. The clinical presentation is mainly related to the tumor location [5]: suprasellar lesions cause diabetes insipidus (DI), hypopituitarism, or bilateral temporal hemianopsia, whereas lesions of the pineal region produce signs of increased intracranial pressure (ICP) due to obstructive hydrocephalus, Parinaud's syndrome, ataxia, behavioral changes, and seizure. The time of diagnosis is related to the presenting symptoms: signs of increased ICP are associated to earlier diagnosis, while endocrine dysfunction or behavioral alteration is associated with delayed diagnosis [6]. Despite specific CT and MRI features for each GCT, histologic types have been described in literature [7, 8], and these tumors are associated with increased markers such as alpha-fetoprotein (AFP), beta-human chorionic gonadotropin (beta-hCG), and placental alkaline phosphatase (PLAP), a definitive histologic diagnosis is necessary to plan proper treatment [9–11]. Mature teratomas are treated with surgical resection, while other GCT needs a combination of surgery, radiotherapy, and chemotherapy according to the tumor type [12, 13]. Germinoma and mature teratomas have the best prognosis.

## 2. Case Presentation

**Figure 1:**
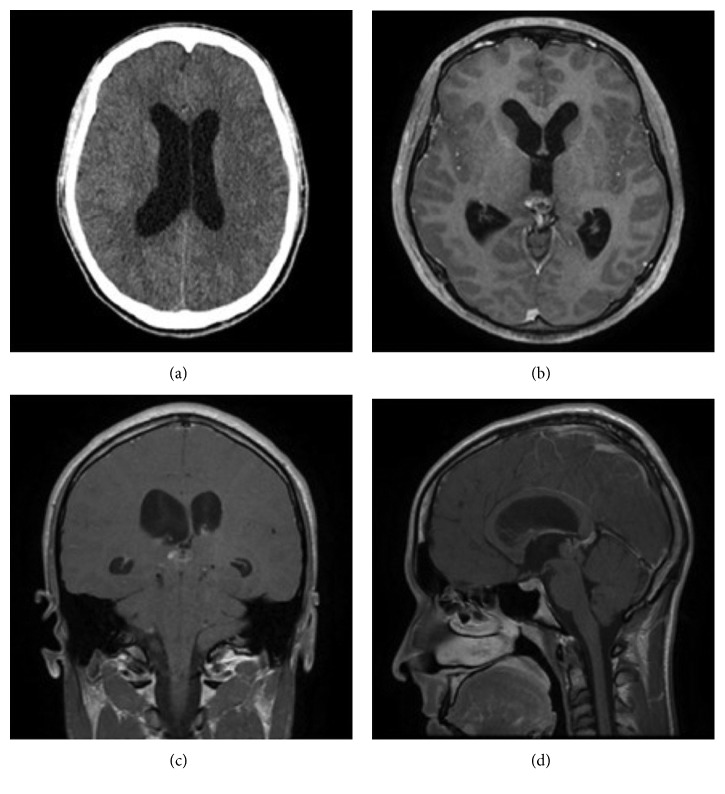
CT scan and MRI at admission documented acute obstructive hydrocephalus caused by a pineal region mass.

A 20-year-old male was admitted to our department in January 2017 with a four-day history of headache and drowsiness. The computed tomographic (CT) scan and magnetic resonance imaging (MRI) demonstrated acute obstructive hydrocephalus due to a mass in the pineal region (maximum diameter of 1.5 cm) with signs of previous hemorrhage and without significative contrast enhancement (Figure 1). An external ventricular drain (EVD) was positioned with neurological improvement.

**Figure 2:**
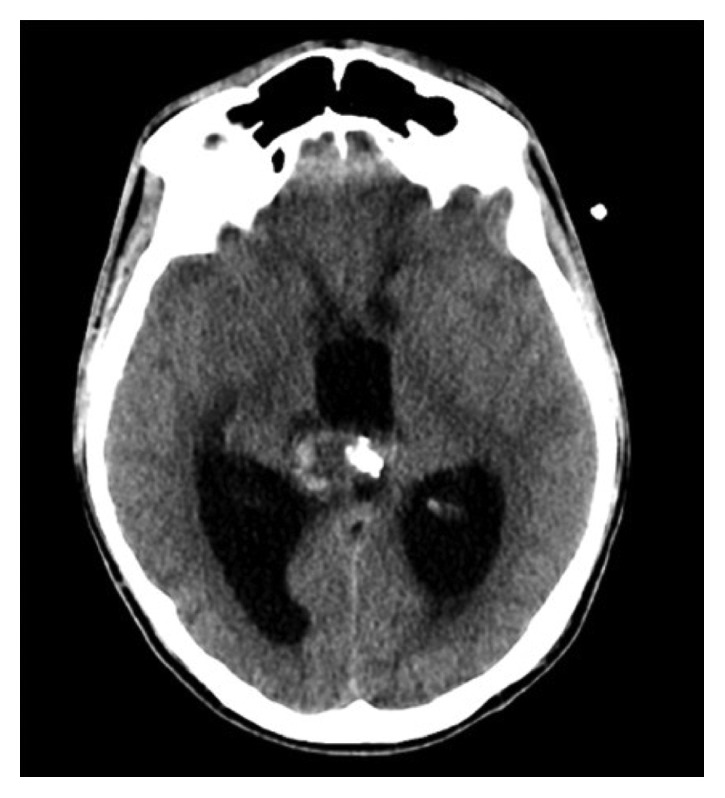
Acute obstructive hydrocephalus 24 hours after EVD closure.

The serum and CSF levels of beta-hCG were 215 IU/L and 447 IU/L, respectively; the serum and CSF levels of AFP were within normal range. The full-body CT scans, spine MRI, and testicular ultrasounds were negative. Tumors of the pineal region may vary from benign to malignant lesions and are classified into four categories: GCT, pineal cell tumors (such as pineocytoma, pinealoblastoma), glial cell tumors, and miscellanea tumors [14, 15]. In this patient, the lesion site, his age, and tumor marker levels strongly suggested a GCT. Given the high level of beta-hCG and normal level of AFP, a germinoma, choriocarcinoma, or mixed type tumor was taken into account. The patient underwent endoscopic third ventriculostomy (ETV) with biopsy of the anterior part of the lesion which appeared as a purplish and friable mass in the posterior part of the third ventricle; a good hemostasis was obtained without evidence of intraventricular and/or mass hemorrhage. EVD was left in place. The histological exam of the surgical specimen documented neural tissue with ependymal cells, macrophages, and cells of germ line without neoplastic elements. The CT scan four days after surgery demonstrated no signs of hemorrhage. On the fifth day after surgery, EVD was closed but 24 hours later, the patient showed neurological worsening with drowsiness and cognitive function decline; thus, EVD was reopened (Figure 2).

**Figure 3:**
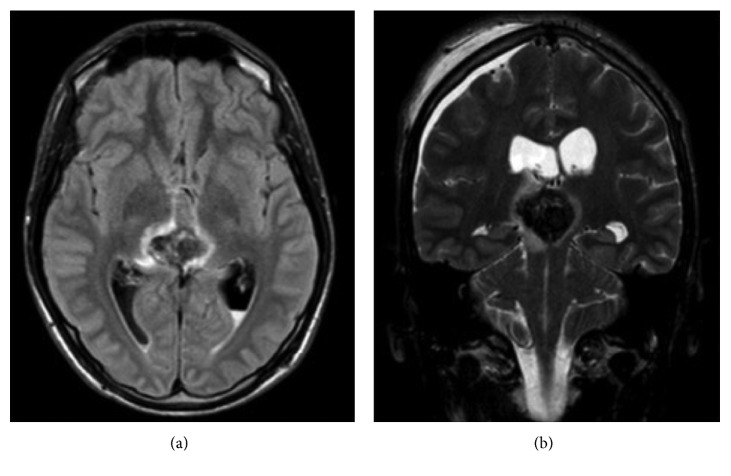
Intratumoral hemorrhage with mass expansion and complete occlusion of the third ventricle.

**Figure 4:**
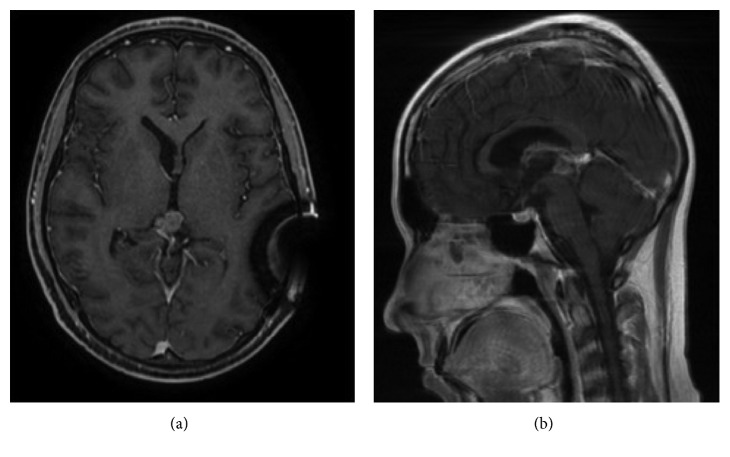
MRI after chemotherapy shows residual tumor mass. For this lesion, the patient underwent stereotactic radiosurgery.

**Figure 5:**
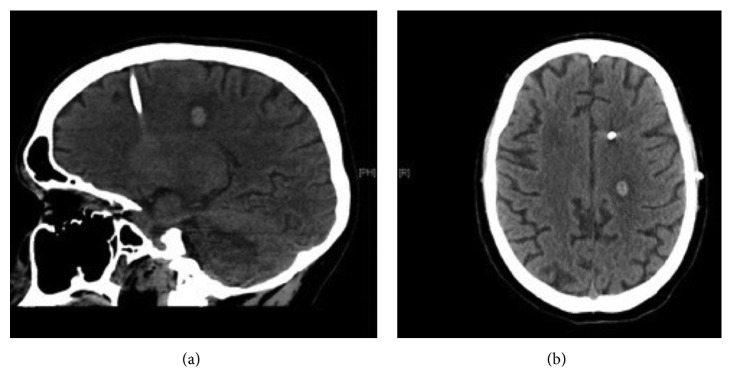
CT scan showing the new cerebral focal lesion.

**Figure 6:**
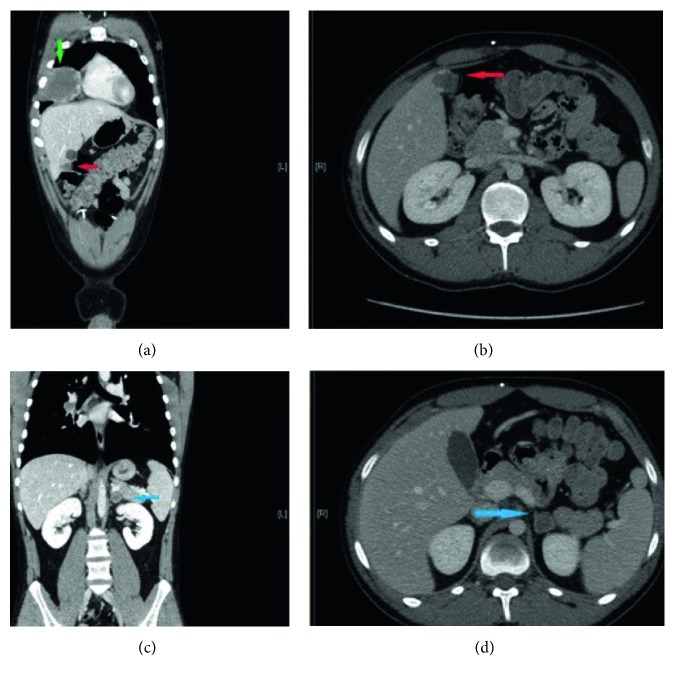
CT scan of the thorax and abdomen showing the lung (green arrow), liver (red arrow), and left adrenal (blue arrow) lesions.

Because of the onset of bacterial meningitis (*Staphylococcus haemolyticus* isolated from CSF specimen), EVD was substituted and clinical picture improved with antibiotic therapy after ten days. MRI on the twelfth day after surgery demonstrated intratumoral hemorrhage with significative mass expansion, with blood in the third ventricle, and with mesencephalic aqueduct (Figure 3). The ventricoloperitoneal (VP) shunt was positioned one month later after complete infection recovery. The patient underwent four cycles of bleomycin/etoposide/cisplatin (BEP) regimen, and the MRI at four months from surgery (Figure 4) showed reduction of mass volume and normal size of brain ventricles. After four cycles of chemotherapy, beta-hCG serum level decreased, but complete normalization was not obtained (serum level was 26 vIU/L). The neurologic status after treatment of hydrocephalus and first-line chemotherapy was good and patient underwent stereotactic radiosurgery for the residual mass. Although in nongerminomatous GCTs the whole brain radiotherapy would be indicated, the patient, also for the absence of histologic diagnosis, refused a whole irradiation due to its high morbidity compared to the stereotactic radiosurgery. Anyway, two months later, he started to complain general malaise and gait imbalance, and the full-body CT scan showed increased intracerebral tumor mass with a new focal lesion and systemic metastasis involving the lungs, liver, and adrenal gland (Figures 5 and 6); the serum levels of beta-hCG were 12.713 UI/L. A liver biopsy was obtained, but no malignant cells were found.

A second line of chemotherapy was started with partial decrease of serum beta-CGH until 1.883 UI/L without any decrease of cerebral or systemic lesions. At the present, after 10 months from admission, the patient is still alive with a poor prognosis.

## 3. Discussion

**Figure 7:**
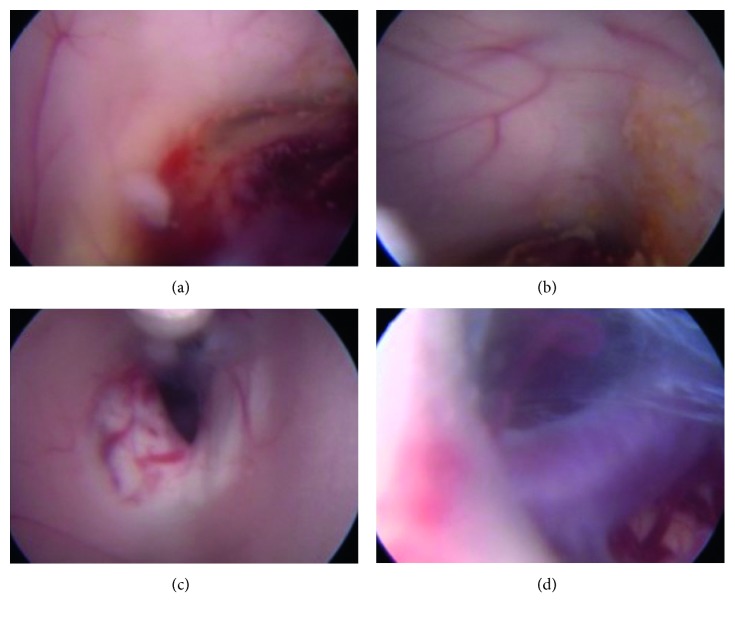
Endoscopic third ventriculostomy procedure. (a) Anterior view of the tumor. (b) Subependymal spread. (c) Stoma. (d) Stoma with basilar artery view.

Taking into account the range of tumors which may occur in the pineal region, histologic diagnosis is necessary to get a correct management. Specimens for diagnosis can be obtained by an open procedure, a stereotactic biopsy or an endoscopic approach [14, 16]. Open procedure is generally avoided because many lesions of the pineal region show high response rates to radiotherapy and/or chemotherapy, so aggressive surgical removal is debated. Despite data from literature confirming that stereotactic biopsy is a safe and effective procedure, endoscopic biopsy (Figure 7) is preferred because it allows both biopsy with direct visualization of the tumor and treatment of obstructive hydrocephalus by third ventriculostomy [17–20]. Moreover, most tumors are mixed type ones and the direct visualization of the lesion allows a multiple sampling that increases the probability of a correct histological diagnosis. It has been suggested that endoscopic biopsy should be reserved only for cases of GCTs with normal serum markers because, in case of marker increase, the histologic exam taken in isolation may lead to incorrect diagnosis of benign lesion if the malignant cells are not present in the specimen [21]. We agree with many authors, and we believe that both markers and histologic diagnosis should be carefully valued to identify the type of tumor even in patients with elevated tumor markers [22]. In patients with increase hCG serum level, some authors prefer to start with chemotherapy without biopsy because of the high diagnostic specificity for choriocarcinoma [23–28]; anyway, the initial serum level of hCG in our case (215 UI/L) was significantly lower than the values reported in literature, so the biopsy was mandatory. It is well known that cerebral GCS has a high probability of hemorrhage during stereotactic, minimally invasive, and endoscopic procedures [29–32]. In our case, we observed hemorrhage between the fifth and the twelfth day after surgery, and so we confirm the high risk of intraventricular hemorrhage of this lesion. We also supposed that the bleeding into the ventricles was the cause of ventriculostomy failure. In this case, the position of the VP shunt is safe even in case of previous CSF infection [33]. This is described also for other internal devices such as spinal and orthopedics prothesis before proper antibiotic therapy [34–36]. Nongerminomatous primary intracranial GCTs are high aggressive tumor with a 3-year survival rate ranging from 27.3% for pure malignant tumor to 70% for mixed type with some elements of pure malignant tumor and both cerebrospinal fluid (CSF) and blood dissemination may occur; metastasis through a VP shunt has also been described [37]. In this case, we supposed a blood dissemination of tumor because of the absence of spinal metastasis, which are related to CSF way, and/or intraperitoneal lesions. Finally, because nongerminomatous GCT has poor prognosis compared with germinoma, whole brain irradiation is required to prevent the relapse either locally or metastatic disease in particular CSF spread. The whole brain radiotherapy treatment has been proposed but patient refused it.

## 4. Conclusion

In cases of pineal region tumor, ETV and biopsy are valid options as primary surgical approach, but, because of the hemorrhage high risk, third ventriculostomy failure may occur, so a careful clinical follow-up is necessary for the prompt positioning of a ventricularperitoneal shunt.

## References

[1] The Committee of Brain Tumor Registry of Japan (2000). Special report of brain tumor registry of Japan (1969–1993). *Neurologia Medico-Chirurgica*.

[2] Matsutani M., Sano K., Takakura K. (1997). Primary intracranial germ cell tumors: a clinical analysis of 153 histologically verified cases. *Journal of Neurosurgery*.

[3] Sugiyama K., Uozumi T., Kiya K. (1992). Intracranial germ-cell tumor with synchronous lesions in the pineal and suprasellar regions: report of six cases and review of the literature. *Surgical Neurology*.

[4] Jennings M. T., Gelman R., Hochberg F. (1985). Intracranial germ-cell tumors: natural history and pathogenesis. *Journal of Neurosurgery*.

[5] Echeverria M. E., Fangusaro J., Goldman S. (2008). Pediatric central nervous system germ cell tumors: a review. *Oncologist*.

[6] Crawford J. R., Santi M. R., Vezina G. (2007). CNS germ cell tumor (CNSGCT) of childhood: presentation and delayed diagnosis. *Neurology*.

[7] Fujimaki T., Matsutani M., Funada N. (1994). CT and MRI features of intracranial germ cell tumors. *Journal of Neuro-Oncology*.

[8] Chang T., Teng M. M., Guo W. Y., Sheng W. C. (1989). CT of pineal tumors and intracranial germ-cell tumors. *American Journal of Roentgenology*.

[9] Allen J. C., Nisselbaum J., Epstein F. (1979). Alphafetoprotein and human chorionic gonadotropin determination in cerebrospinal fluid: an aid to the diagnosis and management of intracranial germ-cell tumors. *Journal of Neurosurgery*.

[10] Haase J., Norgaard-Pedersen B. (1979). Alpha-feto-protein (AFP) and human chorionic gonadotropin (hCG) as biochemical markers of intracranial germ cell tumours. *Acta Neurochirurgica*.

[11] Shinoda J., Yamada H., Sakai N. (1988). Placental alkaline phosphatase as a tumor marker for primary intracranial germinoma. *Journal of Neurosurgery*.

[12] Kyritsis A. P. (2010). Management of primary intracranial germ cell tumors. *Journal of Neuro-Oncology*.

[13] Souweidane M. M., Krieger M. D., Weiner H. L., Finlay J. L. (2010). Surgical management of primary central nervous system germ cell tumors: proceedings from the Second International Symposium on Central Nervous System Germ Cell Tumors. *Journal of Neurosurgery: Pediatrics*.

[14] Regis J., Bouillot P., Rouby-Volot F., Figarella-Branger D., Dufour H., Peragut J. C. (1996). Pineal region tumors and the role of stereotactic biopsy: review of the mortality, morbidity, and diagnostic rates in 370 cases. *Neurosurgery*.

[15] Edwards M. S. B., Hudgins R. J., Wilson C. B., Levin V. A., Wara W. M. (1988). Pineal region tumors in children. *Journal of Neurosurgery*.

[16] Bruce J. N., Ogden A. T. (2004). Surgical strategies for treating patients with pineal region tumors. *Journal of Neuro-Oncology*.

[17] Gangemi M., Maiuri F., Colella G., Buonamassa S. (2001). Endoscopic surgery for pineal region tumors. *Minimally Invasive Neurosurgery*.

[18] Oi S., Shibata M., Tominaga J. (2000). Efficacy of neuroendoscopic procedures in minimally invasive preferential management of pineal region tumors: a prospective study. *Journal of Neurosurgery*.

[19] Morgenstern P. F., Osbun N., Schwartz T. H., Greenfield J. P., Tsiouris A. J., Souweidane M. M. (2011). Pineal region tumors: an optimal approach for simultaneous endoscopic third ventriculostomy and biopsy. *Neurosurgical Focus*.

[20] Iacoangeli M., Colasanti R., Esposito D. (2017). Supraorbital subfrontal trans-laminar endoscope-assisted approach for tumors of the posterior third ventricle. *Acta Neurochirurgica*.

[21] Luther N., Edgar M. A., Dunker I. J., Souweidane M. (2006). Correlation of endoscopic biopsy with tumor marker status in primary intracranial germ cell tumors. *Journal of Neuro-Oncology*.

[22] Oi S., Matsuzawa K., Choi J. U. (1998). Identical characteristics of the patient populations with pineal region tumors in Japan and in Korea and therapeutic modalities. *Child’s Nervous System*.

[23] Romshe C. A., Sotos J. (1975). Intracranial human chorionic gonadotropin-secreting tumor with precocious puberty. *Journal of Pediatrics*.

[24] Ono N., Inoue H. K., Naganuma H., Misumi S., Tamura M. (1986). Germ cell tumor in the basal ganglia: immunohistochemical demonstration of α-fetoprotein, human chorionic gonadotropin, and carcinoembryonic antigen. *Surgical Neurology*.

[25] Tada T., Takizawa T., Nakazato F. (1999). Treatment of intracranial nongerminomatous germ-cell tumor by high-dose chemotherapy and autologous stem-cell rescue. *Journal of Neuro-Oncology*.

[26] Herrmann H. D., Westphal M., Winkler K., Laas R. W., Schulte FJ. (1994). Treatment of nongerminomatous germ-cell tumors of the pineal region. *Neurosurgery*.

[27] Kitanaka C., Matsutani M., Sora S., Kitanaka S., Tanae A., Hibi I. (1994). Precocious puberty in a girl with an hCG-secreting suprasellar immature teratoma. Case report. *Journal of Neurosurgery*.

[28] Nam D. H., Cho B. K., Shin H. J., Ahn H. S., Kim I. H., Wang K. C. (1999). Treatment of intracranial nongerminomatous malignant germ cell tumor in children: the role of each treatment modality. *Child’s Nervous System*.

[29] Vaquero J., Martinez R., Manrique M. (2000). Stereotactic biopsy for brain tumors: is it always necessary?. *Surgical Neurology*.

[30] Shinoda J., Sakai N., Yano H., Hattori T., Ohkuma A., Sakaguchi H. (2004). Prognostic factors and therapeutic problems of primary intracranial choriocarcinoma/germ-cell tumors with high levels of hCG. *Journal of Neuro-Oncology*.

[31] Iaconageli M., Nocchi N., Nasi D. (2016). Minimally invasive supraorbital key-hole approach for the treatment of anterior cranial fossa meningiomas. *Neurologia Medico-Chirurgica*.

[32] Sun J., Huang Y. (2015). One case of choriocarcinoma sellar region metastasis and literature review. *Chinese Neurosurgical Journal*.

[33] Tunkel A. R., Hartman B. J., Kaplan S. L. (2004). Practice guidelines for the management of bacterial meningitis. *Clinical Infectious Diseases*.

[34] Dobran M., Iacoangeli M., Nasi D. (2016). Posterior titanium screw fixation without debridement of infected tissue for the treatment of thoracolumbar spontaneous pyogenic spondylodiscitis. *Asian Spine Journal*.

[35] Hunter G. A. (1979). The results of reinsertion of a total hip prosthesis after sepsis. *Journal of Bone and Joint Surgery. British Volume*.

[36] Boyle T. A., Uslan D. Z., Prutkin J. M. (2017). Reimplantation and repeat infection after cardiac-implantable electronic device infections: experience from the MEDIC (Multicenter Electrophysiologic Device Infection Cohort) database. *Circulation: Arrhythmia and Electrophysiology*.

[37] Rickert C. H., Reznik M., Lenelle J., Rinaldi P. (1998). Shunt-related abdominal metastasis of cerebral teratocarcinoma: report of an unusual case and review of the literature. *Neurosurgery*.

